# Expression analyses of *CUP-SHAPED COTYLEDON* and *SHOOT MERISTEMLESS* in the one-leaf plant *Monophyllaea glabra* reveal neoteny evolution of shoot meristem

**DOI:** 10.1038/s41598-024-62049-4

**Published:** 2024-05-15

**Authors:** Shunji Nakamura, Ayaka Kinoshita, Hiroyuki Koga, Hirokazu Tsukaya

**Affiliations:** Department of Biological Sciences, Graduate School of Science, The University of Tokyo, Tokyo, 113-0033 Japan

**Keywords:** Morphogenesis, Plant evolution

## Abstract

The one-leaf plant *Monophyllaea glabra* exhibits a unique developmental manner in which only one cotyledon continues growing without producing new vegetative organs. This morphology is formed by specific meristems, the groove meristem (GM) and the basal meristem (BM), which are thought to be modified shoot apical meristem (SAM) and leaf meristem. In this study, we analysed the expression of the organ boundary gene *CUP-SHAPED COTYLEDON* (*CUC*) and the SAM maintenance gene *SHOOT MERISTEMLESS* (*STM*) orthologs by whole-mount in situ hybridisation. We found that *CUCs* did not show clear border patterns around GM and BM during the vegetative phase. Furthermore, double-colour detection analysis at the cellular level revealed that *CUC* and *STM* expression overlapped in the GM region during the vegetative phase. We also found that this overlap is dissolved in the reproductive phase when normal shoot organogenesis is observed. Since co-expression of these genes occurs during SAM initiation under embryogenesis in Arabidopsis, our results demonstrate that GM is a prolonged stage of pre-mature SAM. Therefore, we propose that neotenic meristems could be a novel plant trait acquired by one-leaf plants.

## Introduction

Plants have the ability to develop most of their organs post-embryonically and generate new organs throughout their lives, unlike animals in which organogenesis completes during embryogenesis^1^. Flowering plants maintain a source of undifferentiated stem cells through the aboveground-shoot and underground-root systems. These systems are mainly derived from the shoot apical meristem (SAM) and the root apical meristem. SAM generates indeterminate vegetative growth of the stem and produces determinate lateral organs: leaves and their modified forms, namely floral organs.

In typical dicotyledonous plants, embryogenesis is classified into a morphogenesis phase with high cell division and differentiation activity, and a maturation phase in which proliferation is ceased^2^. The morphogenesis phase begins immediately after fertilisation and continues through the globular, early-heart to late-heart stages. Embryogenesis ends after bent cotyledon stages in the maturation phase. SAM becomes histologically visible during embryogenesis with a typical three-layered structure between cotyledons^3^. This typical three-layered structure, evidence of SAM formation, is established during transition from the late globular to the torpedo stage^3^.

The formation of SAM between predetermined cotyledon regions and establishing boundaries between organs are controlled by NAM/ATAF/CUC (NAC) family transcription factors^4–6^. In *Arabidopsis thaliana*, these developmental processes are performed by *CUP-SHAPED COTYLEDON1* (*CUC1*), *CUC2* and *CUC3*, which encode NAC domain proteins^4,6–9^. In *cuc1 cuc2* double mutant seedlings lack embryonic SAM and fuse cotyledons^4^. In SAM initiation and boundary establishment, *SHOOTMERISTEMLESS* (*STM*), which encodes a class I KNOTTED1-LIKE HOMEOBOX (KNOX1) transcription factor, is required in addition to CUC1 and CUC2^8,10^. At the globular embryo stage, the expression domains of *CUCs* and *STM* genes largely overlap in the predetermined cotyledon boundary region^6,8^. CUCs and STM can bind to their respective promoter regions and mutually promote their expression^8,11–13^. During embryogenesis, STM promotes expression of *CUCs*, and also represses *CUC1/2* by indirectly activating microRNA164 that targets CUC1/2 mRNA^11,12,14–16^. As a result, a boundary region is formed by suppressing the expression of *CUC* in the region where *STM* is expressed. Thus, expression of *CUCs* is excluded from the centre of the STM-expressing region, and *STM* expression is restricted to the centre of this region at the heart embryo stage. The *STM*-expressed region develops into domed-shape SAM and the growth of the *CUC*-expressed region is suppressed, resulting in a furrow. Therefore, separation of *CUC* and *STM* expression patterns is crucial to the formation and maintenance of SAM.

In Gesneriaceae, all members of the genus *Monophyllaea* and some members of the genus *Streptocarpus* are known to be one-leaf plants^17^. *Monophyllaea* and *Streptocarpus* are not sister genera, although their morphological structures are similar^17–20^. One-leaf plants exhibit a unique developmental manner unlike that of typical seed plants such as the model plant *A. thaliana*^17,21^. One-leaf plants develop two identical cotyledons after germination (called the isocotyledonous stage). Next, one cotyledon (the microcotyledon) stops growing, whereas the other (the macrocotyledon) continues growing (called the anisocotyledonous stage) as a result of competition between them^17,22^. A unique phenotype of one-leaf plants is the lack of new vegetative organ production.

One-leaf plants have a unique shoot-like structure called a phyllomorph that is composed of an indeterminately growing lamina and one petiolode (a stem- and petiole-like organ)^21^. The phyllomorph has three meristems: the groove meristem (GM), the basal meristem (BM) and the petiolode meristem (PM). PM is located at the base of the midrib and is thought to contribute to growth of the midrib and petiolode^21,23,24^. GM and BM are responsible for the unique characteristics of one-leaf plants: indeterminately expanding leaf lamina without producing additional vegetative organs^21,22,24–26^. In histological studies, GM has been suggested to be a modified SAM since it is located at the base of cotyledons, though it lacks a typical dome-like structure in the vegetative stage. However, GM contributes to the differentiation of inflorescence meristems (IMs) after floral induction, and is seemingly silenced in the vegetative phase^21,24,25^. In the reproductive phase, the inflorescence that develops from IMs has the typical shoot structure of angiosperms^23–25^. Meanwhile, two BMs located at the base of macrocotyledons contribute to indeterminate leaf lamina growth, suggesting they are modified leaf meristem^21,22,24,26^.

In *Monophyllaea*, we previously reported that in GM, homologs of not only SAM-specific genes such as *STM*, but also non-SAM-expressed genes such as *ANGUSTIFOLIA3* (*AN3*)/*Arabidopsis thaliana GRF-INTERACTING FACTOR1* (*AtGIF1*) are expressed^27^. *STM* expression is detected in the GM region by tissue-specific laser dissection and in situ hybridisation during the vegetative phase, suggesting that the GM has SAM properties^26,27^. *AN3* is specifically expressed in leaf meristem that is established in the basal part of leaf primordia and regulates cell division in *A. thaliana* and rice^28–32^. Because *AN3* is expressed not only in the BM region but also in the GM region, GM also has lateral organ properties, unlike SAM of usual angiosperms. The mixed expression of the leaf meristem gene and the SAM gene suggests that the boundary region between GM and BM may be ambiguous.

In this study, we identified organ boundary gene *CUC* orthologs of *Monophyllaea glabra* to evaluate the properties of one-leaf plant-specific meristems, especially GM. We compared the expression patterns of *CUCs* with those *STMs* using whole-mount in situ hybridisation (WISH) in different developmental stages. Our findings reveal novel properties related to the maintenance and initiation of meristems in one-leaf plants of the genus *Monophyllaea*.

## Results

### Expression pattern of *Mg-CUCs* in *M. glabra* during the vegetative phase

**Figure 1:**
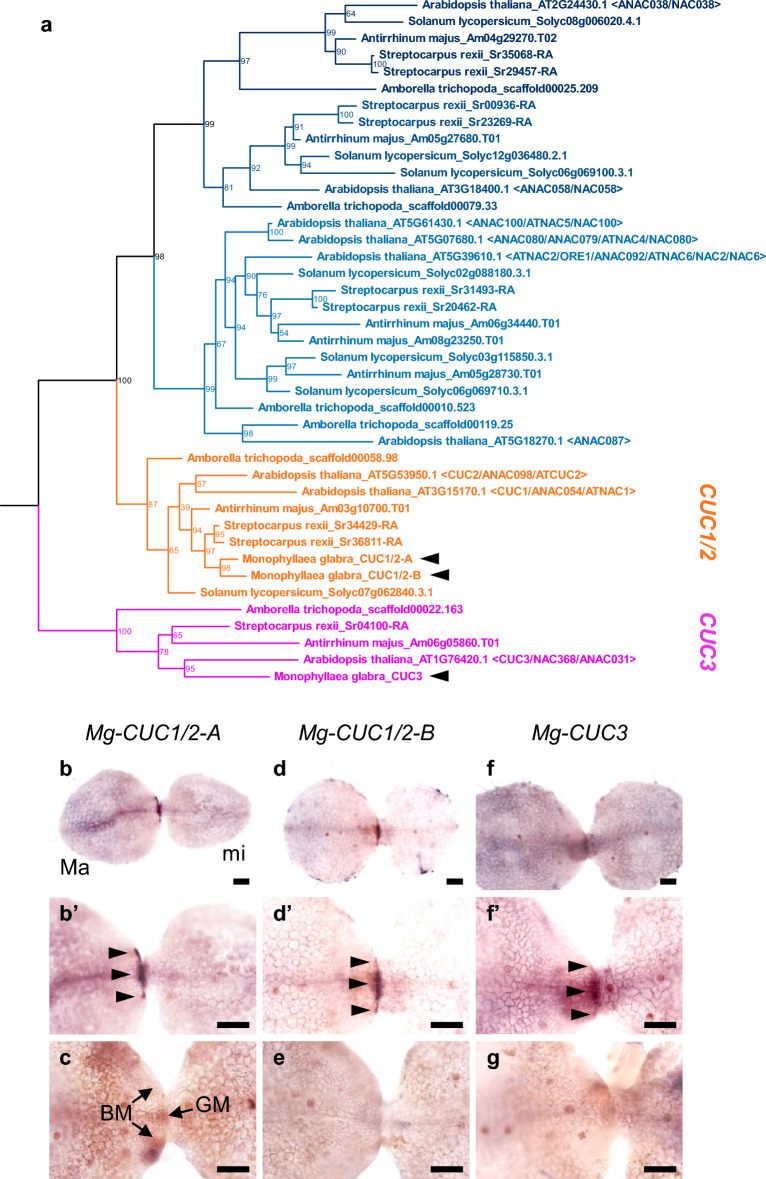
*Mg-CUCs* expression patterns in *Monophyllaea glabra* during the vegetative phase. (**a**) Phylogenetic tree of NAC proteins. Numbers at nodes indicate confidence values (%) calculated from 1000 bootstraps. (**b**–**g**) Whole-mount in situ hybridisation of *Mg-CUCs* at the anisocotyledonous stage: 12 days after sowing (DAS). (**b**,**c**) *Mg-CUC1/2-A*. (**d**,**e**) *Mg-CUC1/2-B*. (**f**,**g**) *Mg-CUC3*. (**b**′,**d**′,**f**′) Magnified images in (**b**,**d**,**f**). (**b**,**d**,**f**) Antisense probes. (**c**,**e**,**g**) Sense probes. Black bars represent 100 μm. *Ma* macrocotyledon, *mi* microcotyledon, *GM* groove meristem, *BM* basal meristem.

To examine the characteristics of specific meristems, we first identified organ boundary gene *CUC* orthologs in *M. glabra*. The phylogenetic relationship in NAC family-related genes from several angiosperms identified two groups, CUC1/2 and CUC3, consistent with previous studies (Fig. 1a)^33^. We identified two *CUC1/2* orthologs, *Mg-CUC1/2-A* and *Mg-CUC1/2-B*, and one *CUC3* ortholog, *Mg-CUC3*. The phylogenetic relationship suggested that *Mg-CUC1/2-A* and *Mg-CUC1/2-B* are paralogs that split in the lineage to *Monophyllaea*. CUCs of *M. glabra* possess an NAC domain that includes highly conserved amino acid motifs (Supplementary Fig. 1)^34,35^. We then isolated their cDNAs to measure gene expression using WISH.

We performed WISH at the early anisocotyledonous stage in which GM and BM were identifiable in the macrocotyledon (Fig. 1b–g). We detected *CUC* expression in the basal region of macrocotyledons, and although there were differences in the degree of staining, these *CUC* expression patterns were similar for each probe of *CUC1/2* and *CUC3* (Fig. 1b–g). Expression of *CUCs* was observed near the BM region, with a spot-shaped character rather than a border pattern (Fig. 1b,d,f). Given previous data on the expression region of *AN3* in BM^27^, we assumed that some *CUC*-expressed cells are included in BM. Interestingly, expression of *CUCs* was also observed in the GM region regarded as modified SAM, indicating that the expression pattern of *CUCs* differed from that of vegetative SAM of *A. thaliana* (Fig. 1b,d,f).

### Expression profiles of *Mg-CUC* and *Mg-STM* around the basal area of the macrocotyledon

**Figure 2:**
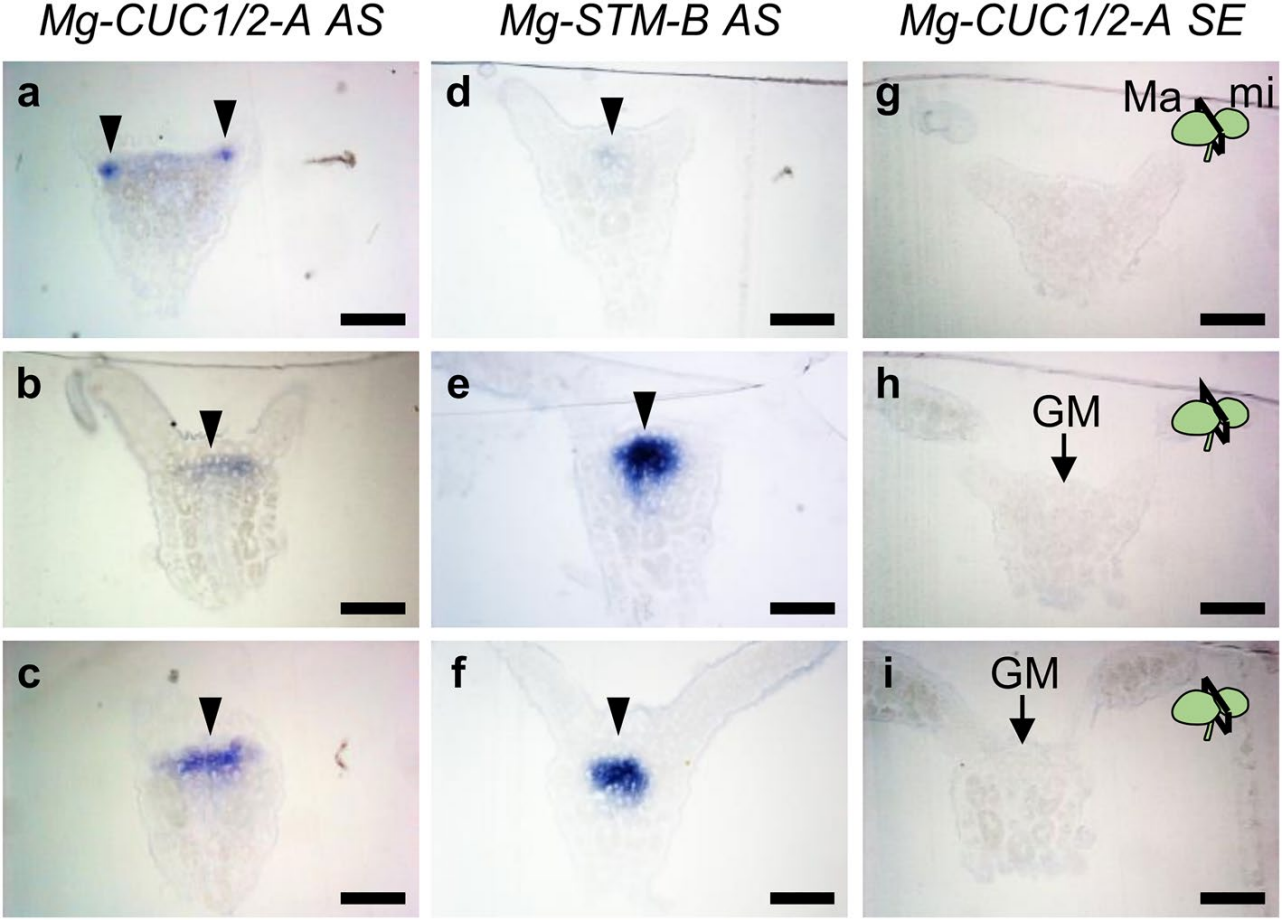
Expression patterns of *CUC* and *STM* orthologs around the basal area of the macrocotyledon. (**a**–**i**) Cross-sections of whole-mount in situ hybridisation samples of *Mg-CUC1/2-A* and *Mg-STM-B* at the anisocotyledonous stage (12 DAS). Examples of the GM region were cross-sectioned from (**a**,**d**,**g**) to the base (**c**,**f**,**i**) of macrocotyledon. (**a**–**c**) Antisense (AS) probes of *Mg-CUC1/2-A*. (**d**–**f**) AS probes of *Mg-CUC1/2-B*. (**g**–**i**) Sense probes (SE) of *Mg-CUC1/2-A*. Black bars represent 100 μm. Black arrowheads indicate gene expression. *Ma* macrocotyledon, *mi* microcotyledon, *GM* groove meristem.

We next assessed cross-section WISH samples to obtain detailed information on *CUC* ortholog expression patterns (Fig. 2). These cross-section samples were prepared from specimens used for gene expression by WISH (Fig. 1b). Because three *CUCs* showed similar gene expression patterns (Fig. 1b–g), we used the probe for *Mg-CUC1/2-A* as a representative in subsequent analysis of *CUC* expression (Fig. 2a–c). In addition, to identify the location of the GM, we detected the expression of *STM* using a probe for the *Mg-STM-B* ortholog expressed in the GM (Fig. 2d–f)^27^. By making serial cross-sections from the tip of the macrocotyledon to the microcotyledon, we sequentially observed the BM region, the region between the BM and GM, and the GM region (see Fig. 1d). Expression of *CUC* was confirmed near both BM regions of the macrocotyledon, where *STM* expression was hardly observed (Fig. 2a,d,g), and was also spot-like rather than border-like in cross-sections, indicating that *CUC* expression near the BM was restricted to a small region (Figs. 1d and 2a). *CUC* expression was also detected in the GM region around the basal area of the macrocotyledon, covering a slightly wider and shallower area than that of the *STM* expression domain (Fig. 2b,c,e,f). It should be noted that *CUC* and *STM* were expressed in the same cells of the subepidermal tissue of GM (Fig. 2b,e). These results demonstrate that the *CUC1/2* expression pattern observed in GM was different from that observed in SAM of typical seed plants such as *A. thaliana*.

### Simultaneous detection of *Mg-CUC* and *Mg-STM* expression by double-fluorescent signals

**Figure 3:**
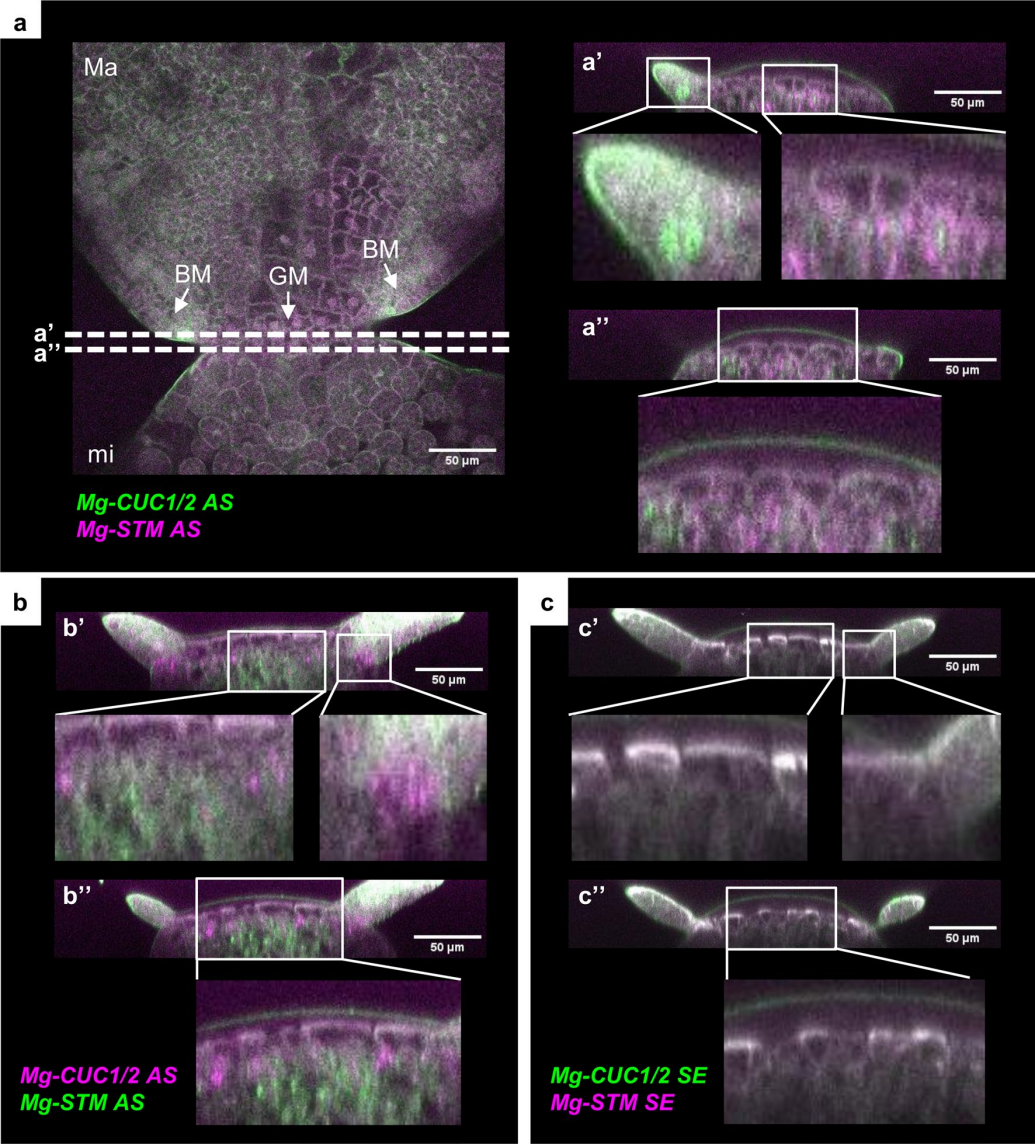
Detection of double gene expression for *CUC* and *STM* orthologs by whole-mount fluorescent in situ hybridisation. (**a**–**c**) Whole-mount fluorescent in situ hybridisation of *Mg-CUC1/2-A* and *Mg-STM-B* at the anisocotyledonous stage (12 DAS). (**a**) Antisense probes of *Mg-CUC1/2-A* (green) and *Mg-STM-B* (magenta). Serial optical sections of a *M. glabra* seedling after double-detection with antisense probes. Cross-sectional images were constructed by Fiji and are shown in (**a**′) and (**a**″). The positions of the two sections (**a**′ and **a**″) are shown by the white dotted line. (**b**) Antisense probes of *Mg-STM-B* (green) and *Mg-CUC1/2-A* (magenta). Cross-sectional images are shown in (**b**′) and (**b**″). (**c**) Sense probes of *Mg-CUC1/2-A* (green) and *Mg-STM-B* (magenta). Cross-sectional images are shown in (**c**′) and (**c**″). Cell walls stained (grey) with Calcofluor White. *Ma* macrocotyledon, *mi* microcotyledon, *GM* groove meristem, *BM* basal meristem.

To analyse the localisation of *CUC* and *STM* expression in the same tissue at higher resolution, we developed a double-colour whole-mount fluorescent in situ hybridisation (WM-FISH) system for *M. glabra* (Fig. 3). In the WM-FISH system, simultaneous detection using fluorescein isothiocyanate (FITC) and digoxigenin (DIG)-labelled probes allowed us to obtain the spatial cellular localisation information for two different genes. In addition, we stained the cell wall with calcofluor white to visualise the localisation of gene expression at cellular-level resolution. Using this system, we analysed the expression patterns of *CUC* and *STM* orthologs during the vegetative stage of *M. glabra* (Supplementary Fig. 2). To confirm that we were not detecting artificial signals with the probe sets, we prepared two types of probe sets with different combinations of target genes. As shown in Fig. 3a,b, we detected positive FISH signals characterised by aggregated granular dots, which are typical of the tyramide signal amplification system using antisense probes^36^.

*CUC* expression, seen as green signals, was detected near the BM region and in the GM region (Fig. 3a and Supplementary Fig. 2a). Since these expression patterns were consistent with the colorimetric WISH results (Fig. 2), the signals detected with this system likely reflect expression of the target genes. Expression of *CUC* observed near the BM region was restricted to one or two cells, and granular aggregations were detected over the whole cell (Fig. 3a and Supplementary Fig. 2a). On the other hand, for *CUC* expression observed in GM, aggregation of granular signals was detected in part of the cell rather than the whole cell (Fig. 3a and Supplementary Fig. 2a). Staining reflecting *CUC* expression was observed throughout the entire cell by colorimetric WISH (Fig. 2), which may be due to limitations of the experimental system, such as probe penetration into the cell. Alternatively, it may reflect actual subcellular localisation differences or differences in expression levels between tissues. Fluorescence signals for *STM*, seen as magenta signals, were also observed in the same samples where signals for *CUC* were detected (Fig. 3a and Supplementary Fig. 2a). Expression of *STM* was narrower and deeper than that of *CUC* (Fig. 3a and Supplementary Fig. 2a). Expression of these genes overlapped in the second and third layers beneath the epidermis, and *CUC* expression was also confirmed in the surrounding areas (Fig. 3a and Supplementary Fig. 2a). These expression patterns were observed even when we replaced the combination of FITC and DIG probes with target genes (Fig. 3b and Supplementary Fig. 2b). Since the expression patterns of *CUC* and *STM* do not overlap in the post-embryogenesis of *A. thaliana*^8–10,37^, the overlapping expression patterns of these genes may indicate a GM-specific mechanism of morphogenesis in one-leaf plants. *CUC* expression in BM and GM partially or totally overlapped in each meristem rather than in the boundary region between BM and GM, suggesting that *CUCs* of *M. glabra* may not yet exhibit the function as the organ boundary gene, at least during the vegetative phase.

### Observation of transition from GM to floral meristem

The *Monophyllaea* body is composed of a phyllomorph, a shoot structure unique to one-leaf plants in the vegetative phase, but in the reproductive phase the shoot system of the inflorescence follows the typical developmental pattern observed in *A. thaliana*, making lateral meristems and lateral organs from IM and floral meristem (FM). Accordingly, we attempted to examine the gene expression patterns in IM and FM, which are morphologically similar to their counterparts in typical plants.

**Figure 4:**
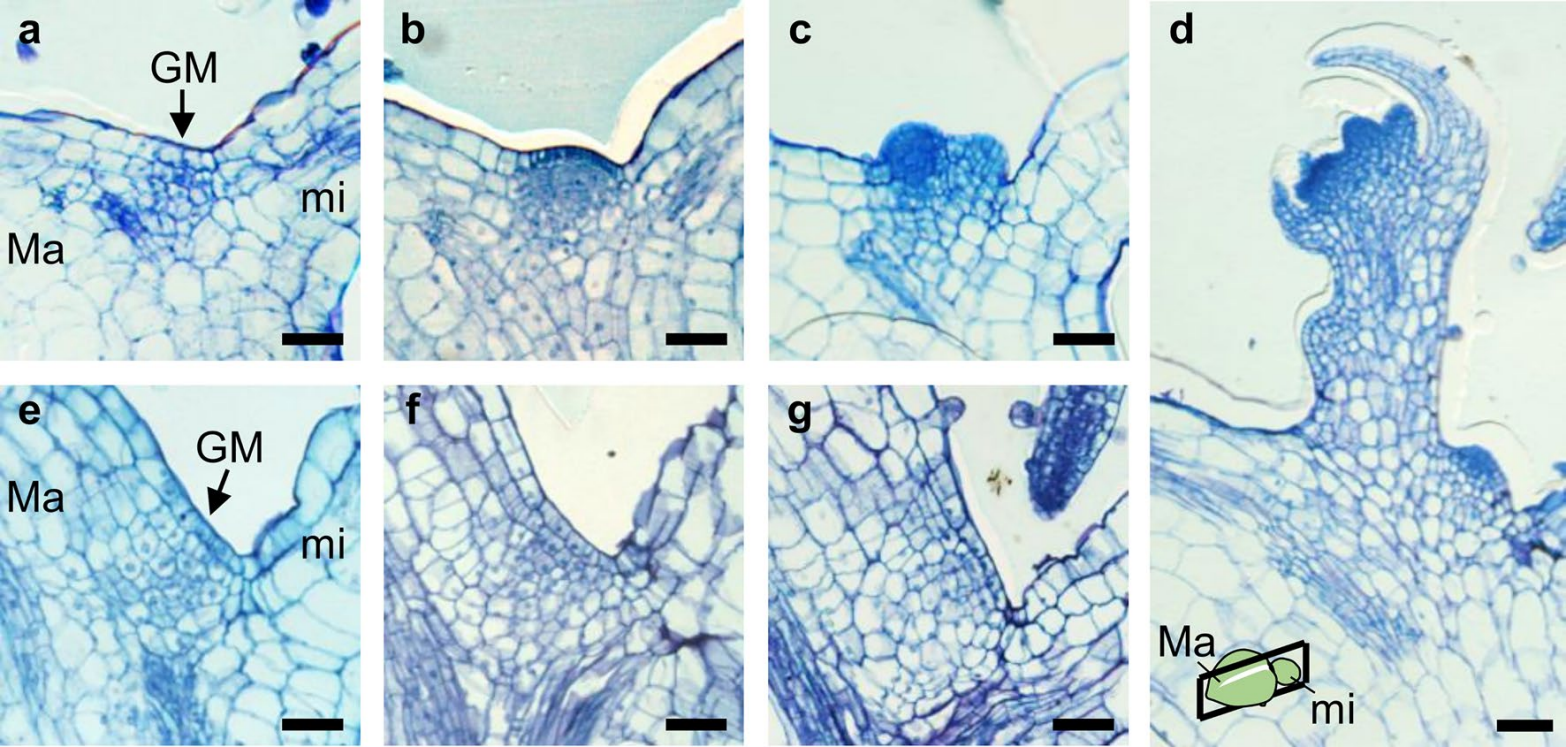
Formation of flower meristem from groove meristem. Development of the groove meristem under short-day conditions (**a**–**d**) and under continuous light conditions (**e**–**g**). (**a**,**e**) 28DAS. (**b**,**f**) 35 DAS. (**c**,**g**) 42 DAS. (**d**) 49 DAS. Black bars represent 50 μm. *Ma* macrocotyledon, *mi* microcotyledon, *GM* groove meristem.

Before examining the expression of *CUC* and *STM* in IM and FM, we first observed the process of meristem formation from GM. GM of *M. glabra* exhibits low cell division activity during the vegetative phase, but accelerated cell division activity after shifting to the reproductive phase, resulting in the formation of GM-derived IM^25,27^. Because *M. glabra* is a short-day plant, and flowering is induced earlier under short-day conditions than under continuous light conditions^17^, we compared the process of IM development under short-day conditions with GM under continuous light conditions (Fig. 4). Under short-day conditions, a dome-shaped structure was formed near GM, and this was IM exhibiting a tunica-corpus structure, which is typical of shoot meristems (Fig. 4b,c)^25^. As shown in Fig. 4d, FM formed thereafter. On the other hand, we observed no such dome-like structure after the same number of days under continuous light conditions (Fig. 4e–g).

### Expression patterns of *Mg-CUC* and *Mg-STM* during the reproductive phase

**Figure 5:**
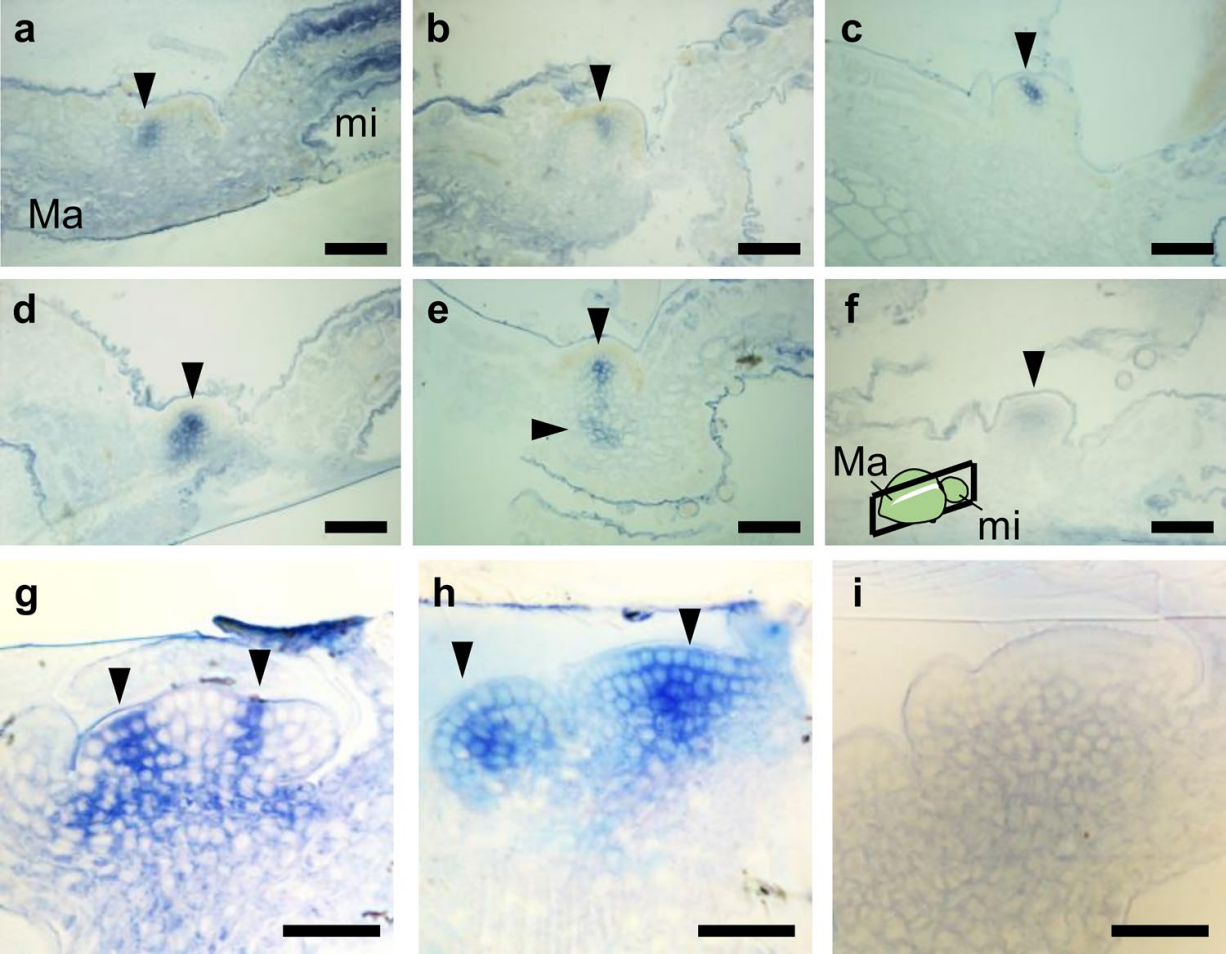
Expression patterns of *CUC* and *STM* orthologs during the reproductive phase. (**a**–**i**) Longitudinal sections of whole-mount in situ hybridisation samples of *Mg-CUC1/2-A* and *Mg-STM-B* during the reproductive phase. (**a**–**c**,**g**) Antisense probes of *Mg-CUC1/2-A*. (**d**–**f**,**h**) Antisense probes of *Mg-CUC1/2-A*. (**i**) Sense probes of *Mg-CUC1/2-A*. (**a**,**b**,**d**,**e**) 36 DAS. (**c**,**f**) 42 DAS. (**g**–**i**) 49 DAS. Black bars represent 100 μm (**a**–**f**) and 50 μm (**g**–**i**). Black arrowheads indicate gene expression. *Ma* macrocotyledon, *mi* microcotyledon.

Next, we performed a timecourse analysis of the expression of *CUC* and *STM* in GM and its derivative meristems (see Fig. 4) after transition to the reproductive phase (Fig. 5). *CUC* initially displayed a band-shaped accumulation in front of the pre-dome structure on the macrocotyledon side, and this expression pattern shifted within the dome structure (IM) as the dome structure formed (Fig. 5a–c). By contrast, *STM* expression was maintained at the centre of the dome structure (Fig. 5d–f). Therefore, expression of *CUC* and *STM* became separated during IM formation. In some samples, during the formation of the dome structure (Fig. 5e), *STM* expression was observed in the basal region (in the petiolode direction) away from the centre of the dome structure. Therefore, *STM* expression might partially retain GM properties at the base, even after IM formation.

Next, we analysed the gene expression patterns of *CUC* and *STM* in FMs (Fig. 5g–i). *CUC* was expressed in the meristem boundary region, and *STM* was expressed in the meristem centre (Fig. 5g,h). This expression pattern was similar to that in SAM and FM of *A. thaliana*, suggesting that CUC and STM have similar functions in IM and FM of *M. glabra* as in *A. thaliana*. Therefore, *CUC* of *M. glabra* likely functions as an organ boundary gene in the reproductive phase.

### Expression patterns of *Mg-CUC* and *Mg-STM* during embryogenesis

**Figure 6:**
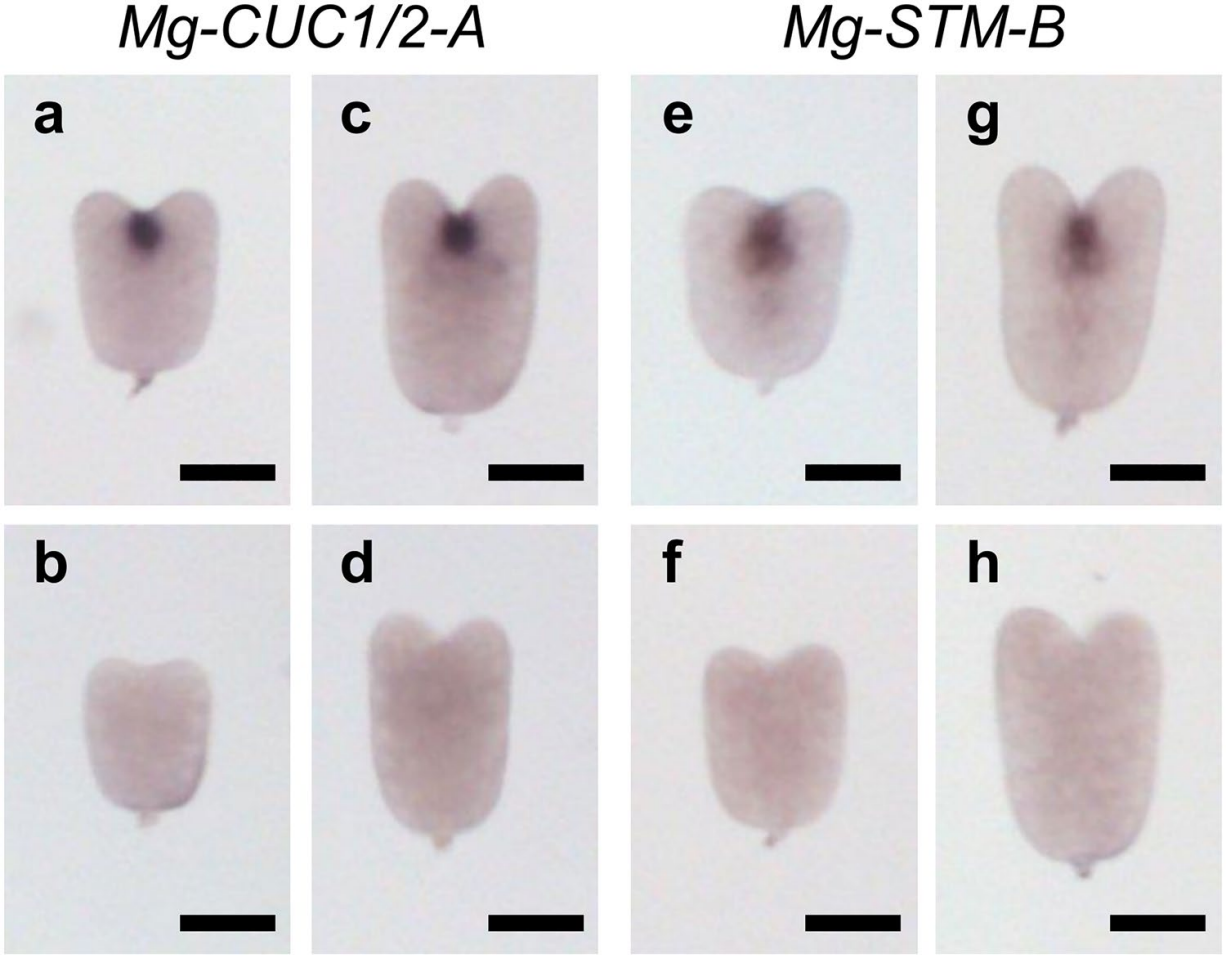
Expression patterns of *CUC* and *STM* orthologs during embryogenesis. (**a**–**h**) Whole-mount in situ hybridisation of *Mg-CUC1/2-A* (**a**–**d**) and *Mg-STM-B* (**e**–**h**) during embryogenesis. (**a**,**c**,**e**,**g**) Antisense probes. (**b**,**d**,**f**,**h**) Sense probes. (**a**,**b**,**e**,**f**) Early heart stage embryos. (**c**,**d**,**g**,**h**) Late heart embryos. Black bars represent 50 μm.

SAM is formed by separating the expression locations of *CUC* and *STM* in *A. thaliana* embryos^8^. Since these expression patterns in *M. glabra* overlapped during the vegetative phase and separated during the reproductive phase, we next assessed the expression of these genes during embryogenesis of *M. glabra*. *CUC* and *STM* expression was observed in cells between the cotyledon primordia in the early heart and late heart stages (Fig. 6). The expression patterns of *CUC* and *STM* observed during embryogenesis were similar to those in GM during the vegetative phase (Figs. 2, 3 and 6). Therefore, it is probable that the expression patterns of *CUC* and *STM* in GM during the vegetative phase are already established during embryogenesis, and they continue during the vegetative phase.

## Discussion

The purpose of the present study was to clarify the characteristics of meristems (in particular, GM) specific to one-leaf plants from the expression pattern of the organ boundary gene *CUC*. We isolated *CUC* orthologs of *M. glabra* and assessed gene expression by WISH (Fig. 1). We found unique expression patterns for *CUC* in GM and BM.

The expression pattern of *CUC* in GM and SAM regions was analysed at various stages of the life cycle of *M. glabra*, including the heart embryo stage, vegetative phase, and reproductive phase (Figs. 1, 2, 5 and 6). *CUC* was expressed between IM and macrocotyledon, and FM and floral organ in the reproductive phase, as seen in authentic SAM of typical angiosperms (Fig. 5). Therefore, the *CUC* ortholog of *M. glabra* appears to function as the organ boundary gene during the reproductive phase. High (~ 90%) amino acid sequence conservation shared with NAC domains of *M. glabra* CUCs further indicates that CUCs perform an organ boundary function also in *M. glabra*.

Many genes expressed in a leaf primordia-specific manner in standard angiosperms including *A. thaliana.* We previously showed that homologs of *PHANTASTICA*, *ROUGH SHEATH2*, *ASYMMETRIC LEAVES1* (*PHAN*/*RS2*/*AS1*) and *AN3* are expressed in the BM region of *M. glabra*^26,27^, indicating a leaf meristem-like identity of BM. Because BM is much more active in cell proliferation than GM in the vegetative stage, and BM region is separated from GM by several enlarged, vacuolated cells^27^, hence something must determine the border between GM and BM. However, in the present study we showed that *CUC*s are not expressed between the GM and BM in a border-like pattern. Therefore, region separation between GM and BM must involve some other border gene(s) in *M. glabra.*

We also previously showed that *AN3* is expressed in both GM and BM^27^. Because GM and BM are thought to be modified SAM and leaf meristem, respectively, expansion of *AN3* expression not only in BM but also in GM suggests incomplete identity separation between BM and GM. This could also be attributed to the absence of *CUC-*dependent border establishment between GM and BM in reproductive phase.

To further test the above possibilities, the cell fates and cell identities must be traced in the GM and BM regions. Because a suitable genetic transformation system is not yet established in *M. glabra,* direct cell file tracing or genetic manipulation are not plausible. However, in the present study, we established WM-FISH to reveal spatial information on expression of specific genes at the cellular level. Using WM-FISH and other approaches such as single-cell transcriptome analysis in combination could trace the detailed cell identity and cell fate changes in meristems of *M. glabra* in the future.

In *A. thaliana*, *CUC* and *STM* are essential genes mediating the formation and maintenance of SAM^4,10^. They are co-expressed in future SAM regions of embryos by mutual activation, then become separately expressed in mature SAM due to indirect inhibition of *CUC* by STM via microRNA164^8,11,16^. However, in *M. glabra*, we showed that expression of *CUC* and *STM* overlaps from embryogenesis until the beginning of the reproductive phase, suggesting a prolonged stage for pre-mature SAM. Indeed, timing of the disappearance of overlapping expression coincides with activation of SAM, resulting in the production of new organs. In *A. thaliana*, foliage-leaf formation was inhibited in transgenic seedlings in which a microRNA164-resistant mutant form of *CUC* was expressed under the control of *STM* regulatory sequences^16^. In other words, if *CUC* and *STM* gene expression regions do not separate, active meristems are not formed. Therefore, we propose that co-expression of *CUC* and *STM* leads to the maintenance of an inactive meristem in the vegetative phase of *M. glabra*. This overlap could be caused by weak suppression of *CUCs* by microRNA164-resistant mutation in *M. glabra*, but we do not support this idea at present because abnormal overlap between *CUCs* and *STM* is only temporally observed in the vegetative phase. Rather, we hypothesise that phase-dependent expression changes in microRNA164 might be a possible mechanism for this unique *CUC*-*STM* overlap in *M. glabra* meristems. Future analysis of microRNA164 in *M. glabra* will test the above idea and could elucidate the GM maintenance mechanism.

From an evolutionary standpoint, we believe that *Monophyllaea* may be the result of neoteny in plants because *M. glabra* GM appears to retain juvenile characteristics of normal SAM. If so, the unique shoot system of one-leaf plants (phyllomorph) could have evolved via prolongation of an immature state, namely neoteny of SAM. Neoteny is a key evolutionary process that triggers neomorph formation and novel body plans in plants and animals^38,39^. Jong and Burt (1975) proposed that the unique one-leaf shoot system in *Streptocarpus* could be due to neoteny, based on the occurrence of inflorescences on cotyledons^21^. Our present interpretation differs from the above idea, focusing on the maturation of SAM formation. We propose that neoteny of meristem, from where new organs are formed, can be possible mechanism underpinning the evolution of novel traits in plants.

## Materials and methods

### Plant materials and growth conditions

Seeds of *Monophyllaea glabra* were originally collected at Strakaew Cave, Thailand under a permission from Mr. T. Wongprasert of the Forest Herbarium, Thailand^26,27^. Voucher specimens are housed in the Forest Herbarium, Department of National Parks, Wildlife and Plant Conservation, Bangkok (BKF), and the University of Tokyo Herbarium (TI). The strain was maintained by cultivation in growth chambers. Plants were grown in plates containing one third Murashige and Skoog salts and 0.8% agar at 22 °C under continuous light or short-day (SD; 8 h light and 16 h dark) conditions. The light intensity was ca. 45 µmol/m^2^/s.

### Isolation and characterisation of *CUC* orthologs from *M. glabra*

Total RNA was extracted from *M. glabra* using an RNeasy plant mini kit (Qiagen, Hilden, Germany) following the manufacture’s protocol. cDNA was reverse-transcribed using a SuperScript III First-Strand Synthesis Kit (Invitrogen, Waltham, MA, USA). Primers for isolating *CUC* homologs were designed based on de novo assembled sequences obtained from mRNA-sequencing (mRNA-seq) data for *M.* *glabra*^27^. Primers for cloning Mg-CUC1/2-A, Mg-CUC1/2-B and Mg-CUC3 were Mg-CUC1/2-A_clon-F1 (5′-ACCTGCACACGCACGCATACTG-3′) and Mg-CUC1/2-A_clon-R1 (5′-ACGGACAGAAACCAGAAATCGGA-3′), Mg-CUC1/2-B_clon-F1 (5′-TGTGTTTCTTGACCTCGCCGGA-3′) and Mg-CUC1/2-B_clon-R1 (5′-GCACTACAGCGATCACGACAAGGT-3′), and Mg-CUC3_clon-F1 (5′-ACGTAGAGTTAAGGCGGGGGA-3′) and Mg-CUC3_clon-R1 (5′-CGTGCTTACTCCATCATCGGGC-3′), respectively. Nucleotide sequences have been deposited in the DNA Data Bank of Japan (DDBJ) (http://www.ddbj.nig.ac.jp/) under accession number LC782938 for *Mg-CUC1/2-A*, LC782939 for *Mg-CUC1/2-B* and LC782940 for *Mg-CUC3*. Although most genes in *M.glabra* would be present in two copies (homeologs)^26,27^, we used one type of isolated gene in this study.

### Molecular phylogenetic analyses

Amino acid sequences from species other than *M. glabra* were obtained from the following databases: Phytozome (https://phytozome-next.jgi.doe.gov/) for *Amborella trichopoda* (version 1.0), *Arabidopsis thaliana* (Araport11), and *Solanum lycopersicum* (ITAG 4.0); Snapdragon Genome Database (http://bioinfo.sibs.ac.cn/) for *Antirrhinum majus* (version 3.0); Plant GARDEN for *Streptocarpus rexii* (version 1.0). Amino acid sequences were aligned by MAFFT^40^, and poorly aligned sequences were trimmed with TrimAL^41^. IQ-TREE2^42^ was used to analyse phylogenetic relationships using the maximum likelihood method. Bootstrap analysis with 1000 replicates was performed using UFBoot2^43^ and phylogenetic trees were visualised using TreeViewer (https://treeviewer.org/).

### Whole-mount in situ hybridisation

cDNA sequences were amplified using primers for target genes. Amplified fragments were cloned into the pZErO-2 vector. Templates for probe synthesis were amplified by PCR using M13 forward (5′-GTAAAACGACGGCCAGT-3′) and M13 reverse (5′-CAGGAAACAGCTATGAC-3′) primers. Using the templates, we prepared DIG-labelled antisense and sense probes with SP6 or T7 polymerases (Roche, Basel, Switzerland) using DIG RNA Labeling Mix (Roche). Whole-mount in situ hybridisation (WISH) was performed as described previously^27,36^. For the WISH, we used embryos at the heart stage and plants at the anisocotyledonous stage in which GM and BM were evident and reproductive stage in which an inflorescence was visible. These were immersed in the fixative solution (4% (w/v) paraformaldehyde with 15% (v/v) dimethyl sulfoxide in phosphate-buffered saline with 0.1% (v/v) Tween-20) for 1 h at room temperature. DIG was detected using a DIG Detection Kit (Roche) with anti-digoxigenin antibody (Roche). Samples were visualised using a MZ16F stereomicroscope (Leica Microsystems, Wetzlar, Germany) or an OM-D E-M10 digital camera (Olympus, Tokyo, Japan) with a SZ61 dissecting microscope (Olympus).

### Histology and microscopy

To observe the formation of reproductive meristem derived from GM, plants grown under continuous light or SD conditions were fixed in formalin-acetic acid-alcohol (FAA) (10% formalin, 5% acetic acid, 50% ethanol (v/v)). The fixed samples were then embedded in Technovit 7100 (Heraeus Kulzer, Wehrheim, Germany) and sliced into 10–15 μm thick sections using a HM360 rotary microtome (Thermo Fisher Scientific, Waltham, Massachusetts, USA). Sections for observation of the transition from GM to floral meristem were then stained with 0.02% (w/v) Toluidine Blue in phosphate-buffered saline containing 0.5% (v/v) Triton X-100 (Nacalai Tesque, Kyoto, Japan). Samples were visualised using a DM4500 light microscope (Leica Microsystems).

### Whole-mount fluorescent in situ hybridisation

FITC-labelled antisense and sense probes were prepared using the same procedure described for the DIG-labelled probe. Whole-mount fluorescent in situ hybridisation was performed using the tyramide amplification system as described previously^44^ with minor modifications. To detect DIG and FITC probes we used mouse anti-digoxigenin antibody IgG1k (Roche) and rabbit anti-FITC polyclonal antibody (Invitrogen) as primary antibodies, respectively. We then used goat anti-mouse antibody with the tyramide amplification system (Alexa Fluor 488 Tyramide SuperBoost Kit, goat anti-mouse IgG; Invitrogen) and goat anti-rabbit antibody with the tyramide amplification system (Alexa Fluor 594 Tyramide SuperBoost Kit, goat anti-rabbit IgG; Invitrogen). We successfully obtained specific granular signals that were typical of the tyramide amplification system in *M. glabra*. The FISH signal in this system reflected the expression of the target gene because we observed no such specific signals when using sense probes (Fig. 3c). Samples were treated with 1% (v/v) Calcofluor White in ClearSee solution^45^ for 1 day to stain the cell walls before visualisation using a FV3000 confocal microscope (Olympus).

### Supplementary Information

Supplementary Figures.

## Data Availability

The datasets presented in this study can be found in online repositories. The datasets of de novo assembled sequences generated during the current study are available from the corresponding author on reasonable request.
