# Supplementary material for: Expression analyses of CUP-SHAPED COTYLEDON and SHOOT MERISTEMLESS in the one-leaf plant Monophyllaea glabra reveal neoteny evolution of shoot meristem
Source: Sci Rep. 2024 May 15;14:11148. doi: 10.1038/s41598-024-62049-4 (PMC11096313; doi:10.1038/s41598-024-62049-4)
Supplement: Supplementary file 1 — Supplementary Figures. [file 41598_2024_62049_MOESM1_ESM.pdf]

Supplementary Information

Expression analyses of *CUP-SHAPED COTYLEDON* and *SHOOT MERISTEMLESS* in the one-leaf plant *Monophyllaea glabra* reveal neoteny evolution of shoot meristem

Shunji Nakamura, Ayaka Kinoshita, Hiroyuki Koga, Hirokazu Tsukaya*

Department of Biological Sciences, Graduate School of Science, The University of Tokyo, Tokyo, 113-0033, Japan
*Correspondence to: tsukaya@bs.s.u-tokyo.ac.jp

This PDF files include:
Supplementary Figures 1 to 2.

```
                              N1                                    N2
AtCUC1          MPPGFRFHPTDEELITYYLLKKVLD---------SNFSCAAISQVDLNKSEPWELPE
AtCUC2          LPPGFRFHPTDEELITHYLLRKVLD---------GCFSSRAIAEVDLNKCEPWQLPG
AtCUC3          LPPGFRFHPTDEELITFYLASKIFH---------GGLSGIHISEVDLNRCEPWELPE
MgCUC1/2-A      LPPGFRFHPLDEELITYYLLKKVLD---------GGFTCRAIAEVDLNKCEPWHLPG
MgCUC1/2-B      MPPGFRFHPLDEELITYYLLKKVLD---------GSFTCRAIAEVDLNKCEPWHLPG
MgCUC3          LPPGFRFHPTDEELITFYLASKVFHTNTRNMMMSSSSSGVQIAEVDLNRCEPWELPE
                 ******** ****** **  *                   *  ****  *** **

                                         N3
AtCUC1          KAKMGEKEWYFFTLRDRKYPTGLRTNRATEAGYWKATGKDREIKSSKTKSLLGMKKT
AtCUC2          RAKMGEKEWYFFSLRDRKYPTGLRTNRATEAGYWKATGKDREIFSSKTCALVGMKKT
AtCUC3          MAKMGEREWYFYSLRDRKYPTGLRTNRATTAGYWKATGKDKEVFSGGGGQLVGMKKT
MgCUC1/2-A      RAKMGEKEWYFFSLRDRKYPTGLRTNRATEAGYWKATGKDREIYSSKTCSLVGMKKT
MgCUC1/2-B      MARMGEKEWYFFSLRDRKYPTGLRTNRATEAGYWKATGKDREIYSSKTCALVGMKKT
MgCUC3          MAKMGEKEWYFFSLRDRKYPTGLRTNRATGAGYWKATGKDREVYSAASGATVGMKKT
                 * *** ****  ***************  ********** *  *       *****

                         N4                              N5
AtCUC1          LVFYKGRAPKGEKSCWVMHEYRLDGKFS-YHYISSSAKDEWVLCKVCLK
AtCUC2          LVFYKGRAPKGEKSNWVMHEYRLEGKFS-YHFISRSSKDEWVISRVFQK
AtCUC3          LVFYKGRAPRGLKTKWVMHEYRLEN----DHSHRHTCKEEWVICRVFNK
MgCUC1/2-A      LVFYLGRAPKGEKSNWVMHEYRLEGKLA-YHYLSRNSKDEWVISRVFQK
MgCUC1/2-B      LVFYMGRAPKGEKSNWVMHEYRLEGKLA-YHYLSRNSKDEWVISRVFQK
MgCUC3          LVFYKGRAPRGLKSKWVMHEYRLHAYLSTSRRYYSSFKEEWVICRIFHK
                **** **** * *  ********              * ***      *
```

Supplemental Figure 1. Structural features of NAC domains of CUCs

The conserved regions in NAC domains of CUCs are shown in the alignment. Highly conserved regions (N1–N5) are boxed. Dashes indicate gaps. Asterisks indicate conserved amino acids. At, *Arabidopsis thaliana*; Mg, *Monophyllaea glabra*.

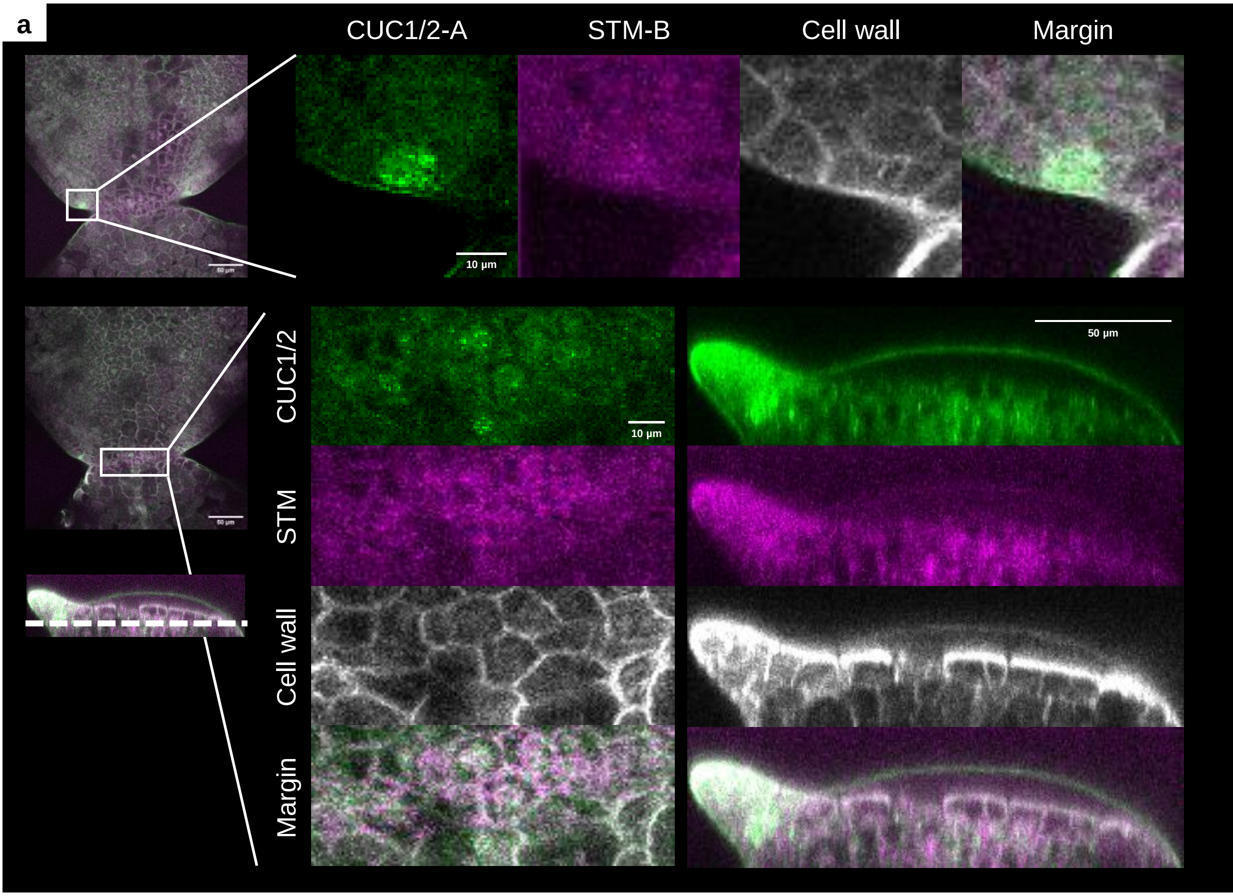

Supplemental Figure 2. Analysis of gene expression for *CUC* and *STM* orthologs using the WM-FISH system (refer to Fig. 3).

(a) Antisense probes of *Mg-CUC1/2-A* (green) and *Mg-STM-B* (magenta). (b) Antisense probes of *Mg-CUC1/2-A* (magenta) and *Mg-STM-B* (green). Serial optical sections of an *M. glabra* seedling after double-detection with antisense probes. Cell walls are stained (grey) with Calcofluor White.

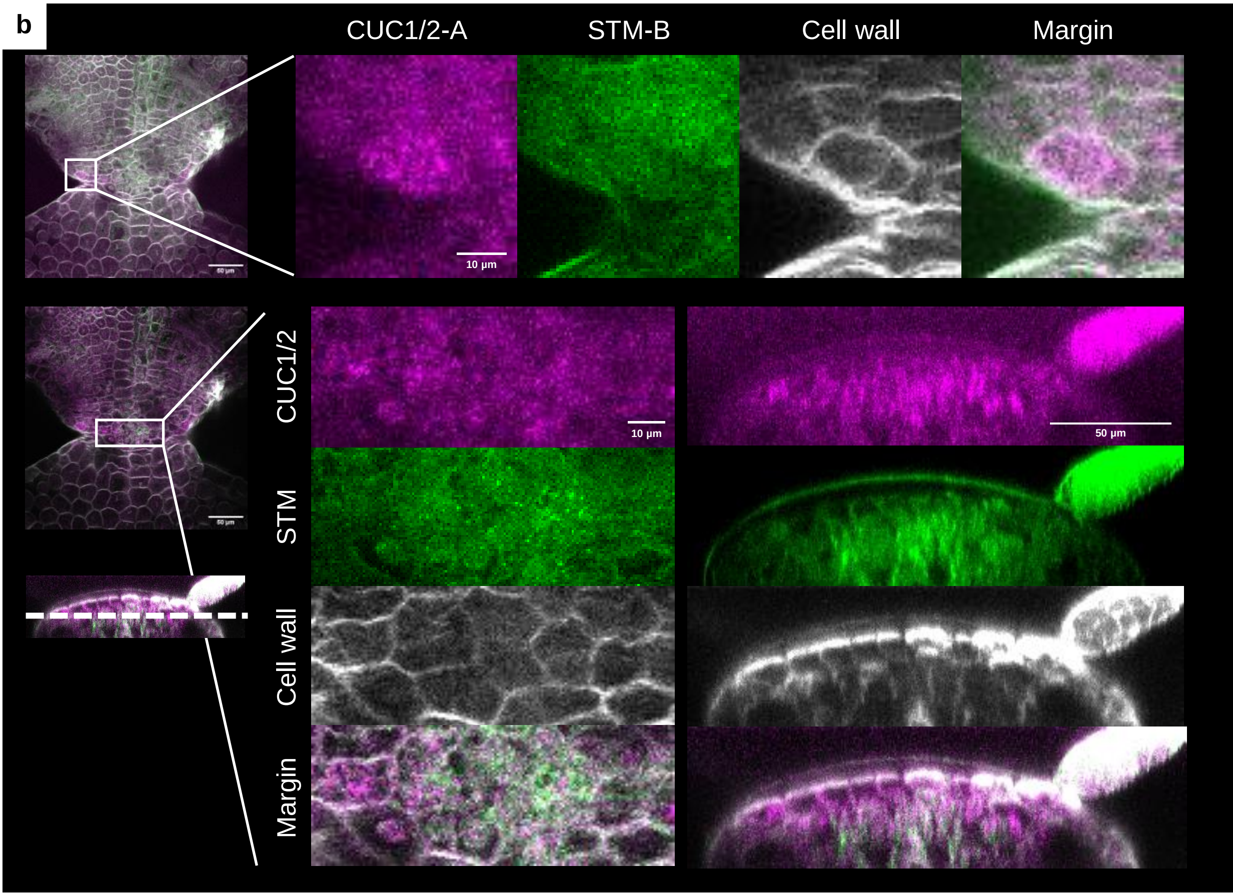

Supplemental Figure 2. Analysis of gene expression for *CUC* and *STM* orthologs using the WM-FISH system (refer to Fig. 3).

(a) Antisense probes of *Mg-CUC1/2-A* (green) and *Mg-STM-B* (magenta). (b) Antisense probes of *Mg-CUC1/2-A* (magenta) and *Mg-STM-B* (green). Serial optical sections of an *M. glabra* seedling after double-detection with antisense probes. Cell walls are stained (grey) with Calcofluor White.
